# Fenofibrate suppresses the progression of hepatoma by downregulating osteopontin through inhibiting the PI3K/AKT/Twist pathway

**DOI:** 10.1007/s00210-023-02604-4

**Published:** 2023-08-11

**Authors:** Weiqing Chen, Feihua Chen, Mouchun Gong, Lijun Ye, Dengcheng Weng, Zhaoqing Jin, Jianjiang Wang

**Affiliations:** Department of General Surgery, First People’s Hospital of Hangzhou Lin’an District, NO.548 Yijin Street, Lin’an District, Hangzhou, Zhejiang 311300 People’s Republic of China

**Keywords:** Fenofibrate, OPN, Twist, HCC

## Abstract

Primary hepatic carcinoma (PHC) is a leading threat to cancer patients with few effective treatment strategies. OPN is found to be an oncogene in hepatocellular carcinoma (HCC) with potential as a treating target for PHC. Fenofibrate is a lipid-lowering drug with potential anti-tumor properties, which is claimed with suppressive effects on OPN expression. Our study proposes to explore the molecular mechanism of fenofibrate in inhibiting HCC. OPN was found extremely upregulated in 6 HCC cell lines, especially Hep3B cells. Hep3B and Huh7 cells were treated with 75 and 100 μM fenofibrate, while OPN-overexpressed Hep3B cells were treated with 100 μM fenofibrate. Decreased clone number, elevated apoptotic rate, reduced number of migrated cells, and shortened migration distance were observed in fenofibrate-treated Hep3B and Huh7 cells, which were markedly abolished by the overexpression of OPN. Furthermore, the facilitating effect against apoptosis and the inhibitory effect against migration of fenofibrate in Hep3B cells were abolished by 740 Y-P, an agonist of PI3K. Hep3B xenograft model was established, followed by treated with 100 mg/kg and 200 mg/kg fenofibrate, while OPN-overexpressed Hep3B xenograft was treated with 200 mg/kg fenofibrate. The tumor growth was repressed by fenofibrate, which was notably abolished by OPN overexpression. Furthermore, the inhibitory effect of fenofibrate on the PI3K/AKT/Twist pathway in Hep3B cells and Hep3B xenograft model was abrogated by OPN overexpression. Collectively, fenofibrate suppressed progression of hepatoma downregulating OPN through inhibiting the PI3K/AKT/Twist pathway.

## Introduction

PHC is one of the most common cancers worldwide, including HCC (75–85%) and intrahepatic cholangiocarcinoma (10–15%) (Sung et al. 2021). Approximately 47% of HCC patients are diagnosed in China, which is the fifth leading cause of death in China (Zhou et al. 2019; Petrick et al. 2020). The main risk factors for HCC are hepatitis virus infection (hepatitis B virus or hepatitis C virus), heavy alcohol consumption, obesity, and autoimmune liver disease. At present, the treatment for HCC is mainly divided into surgical treatment and non-surgical comprehensive treatment. Surgical resection is the first choice for early HCC, however, with a high possibility of relapse. The 5-year recurrence rate is as high as 70% (Forner et al. 2018). Molecular targeted therapy and immunotherapy are optional methods for advanced hepatoma, which are costly with multiple side effects. The median survival time of HCC patients is not more than two years (Yang et al. 2019; Vogel and Saborowski 2020). Therefore, it is urgent to explore effective treatment strategies for HCC.

Osteopontin (OPN) is a phosphorylated glycoprotein that exerts a variety of biological effects by binding to receptors such as integrin and CD44 (Chernaya et al. 2018; Klement et al. 2018), which is involved in pathophysiological reactions such as bone formation, mineralization, reconstruction, inflammatory response, vascular diseases, and the development of tumors (Foster et al. 2018; Lok and Lyle 2019). Previous study has shown that the proliferation, invasion, and metastasis of tumor cells are facilitated by OPN, accompanied by an inhibition on apoptosis (Huang et al. 2017). It is reported that the production of vascular endothelial growth factor is induced by OPN to facilitate the progression of angiogenesis, which further mediates the resistance of cancer cells to chemotherapy (Du et al. 2017; Ouyang et al. 2018). Studies have shown that OPN mediates the occurrence and development of a variety of tumors (Wong et al. 2017; Cao et al. 2019). Furthermore, OPN is significantly upregulated in liver cancer tissues and serum of HCC patients, which plays a critical role in the occurrence, development, metastasis, and recurrence of HCC (Cao et al. 2012). A recent study has confirmed that OPN facilitates the progression of HCC by activating PI3K/AKT/Twist signaling pathway (Yu et al. 2018). Therefore, OPN may become a novel effective target for the treatment of HCC.

Fenofibrate belongs to the third-generation lipid-lowering drugs of phenoxy aromatic acids, which significantly reduces total cholesterol (TC) and total triglyceride (TG) by activating peroxisome proliferator-activated receptor-α (Staels et al. 1998). Fenofibrate is commonly used in the treatment of hypercholesterolemia, hypertriglyceridemia, and mixed hyperlipidemia in clinical practice. Recently, marked anti-tumor effect of fenofibrate has been reported (Kong et al. 2021). However, the mechanism of action remains unclear. The latest study reported that the expression of OPN was suppressed by fenofibrate (Rowbotham et al. 2018). Our study aims to explore the molecular mechanism of fenofibrate in inhibiting the progression of HCC.

## Materials and methods

### Cells and treatments

Normal human hepatocyte cell cline (L-02 cells) and six HCC cell lines (Hep3B cells, HepG2 cells, Huh7 cells, MHCC97H cells, HCCLM3 cells, and HCCLM6 cells) were obtained from iCell (China) and cultured in DMEM medium containing 10% FBS, which were incubated under 37 ℃ and 5% CO2. To obtain OPN-overexpressed cells, Hep3B cells were transfected with adenovirus containing pcDNA3.1-OPN, with pcDNA3.1-NC as a negative control. After 48 h transfection, cells were collected and the transfection efficacy was identified using the Western blotting assay.

### CCK-8 assay

Cells were implanted in 96-well plates for 24 h, followed by adding with 10 μl CCK8 solution. After incubating for 2 h, the OD value was detected using the microplate reader (CMaxPlus, MD, USA).

### Western blotting assay

The BCA kit (pc0020, Solarbio, China) was utilized to quantify the protein isolated from cells, followed by being separated with the 12% SDS-PAGE. The separated protein was transferred from the gel to the PVDF membrane, which was further introduced with 5% skim milk. Then, the membrane was introduced with the primary antibody against PI3K (1:1000, AF6241, Affinity, USA), p-AKT (1:1000, AF0016, Affinity, USA), AKT (1:1000, AF6261, Affinity, USA), OPN (1:2000, AF0227, Affinity, USA), Twist (1:1000, AF4009, Affinity, USA), E-cadherin (1:2000, AF0131, Affinity, USA), N-cadherin (1:1000, AF4039, Affinity, USA), and GAPDH (1:10000, AF7021, Affinity, USA). The second antibody (1:6000, 7074, CST, USA) was subsequently added to be incubated for 90 min. Finally, ECL reagent was added to expose the bands, which were further quantified with the Image J software.

### Wound healing assay

When the cell density reached more than 90%, 200 μL of the gun tip was used to scratch in each well, followed by discarding the medium and replaced with DMEM incomplete medium. Then the scratch in each well was photographed. Cells were put into the incubator, and the scratch of each well was photographed again after 24 h. According to the scratch condition, the 24 h scratch data and the 0 h scratch data were determined. The corresponding scratch width and migration rate was calculated.

### Clone formation assay

Two thousand cells/well were implanted in 6-well plates and incubated for 10 days. When macroscopic cloning appeared, the supernatant was aspirated and cells were fixed with the mixture of methanol and acetic acid at a ratio of 3:1 at room temperature for 5 min. Methanolic solution containing crystal violet was added and fixed for 15 min. The supernatant was aspirated and air-dried at room temperature for observation using an optical microscope (AE2000; Motic, China).

### Transwell assay

The upper chamber of the Transwell insert (3422; Corning, USA) was implanted with 1.5 × 10^5^ cells cultured in serum-free medium, which were then filled with 20% FBS-supplemented medium in the lower chamber. After 24 h incubation, cells were wiped off from the upper chamber, and those in the lower chamber were stained with crystal violet. Finally, the migrated cells were counted using an optical microscope (AE2000; Motic, China).

### The detection of apoptosis using the flow cytometry

1×10^6^ cells were collected and washed by PBS buffer, which were then re-suspended with 300 μl pre-cold 1×Annexin V-FITC binding buffer. Then, cells were introduced with 5 μl Annexin V-FITC reagent and 10 μl PI reagent, followed by 10 min incubation in the dark at room temperature. Lastly, cells were loaded onto the flow cytometry (C6, BD, USA) for the analysis of apoptosis.

### Animals and xenograft model

Twenty-four 24 female nude mice (7–9 week) were purchased from Charles River (China). After 7 days of adaptive feeding, nude mice were randomly divided into 4 groups: Control, 100 mg/kg fenofibrate, 200 mg/kg fenofibrate, and OPN OE+ 200 mg/kg fenofibrate groups. Six nude mice were used in each group. For the establishment of xenograft model, 5*10^6^ cells were inoculated subcutaneously into the back of the axilla per mouse with a volume of 0.25 mL/ mouse. The tumor volume was recorded every three days. The administration was performed until the tumor volume reached approximately 100 mm^3^. In the control group, the Hep3B cell xenograft model was established, followed by orally dosed with normal saline for 14 days. In the 100 mg/kg fenofibrate and 200 mg/kg fenofibrate groups, the Hep3B cell xenograft model was established, followed by orally dosed with 100 mg/kg fenofibrate and 200 mg/kg fenofibrate daily for 14 days, respectively. In the OPN OE+ 200 mg/kg fenofibrate group, the OPN-overexpressed Hep3B cell xenograft model was established, followed by orally dosed with 200 mg/kg fenofibrate daily for 14 days. Tumors were weighed and sampled at the end.

### Statistical analysis

Mean±SD was utilized to present data, which was analyzed using the one-way ANOVA method with the software of GraphPad Prism 7.0 software. *P*<0.05 was considered to be a statistically significant difference.

## Results

### The determination of the HCC cell line and the concentration of fenofibrate

**Fig. 1:**
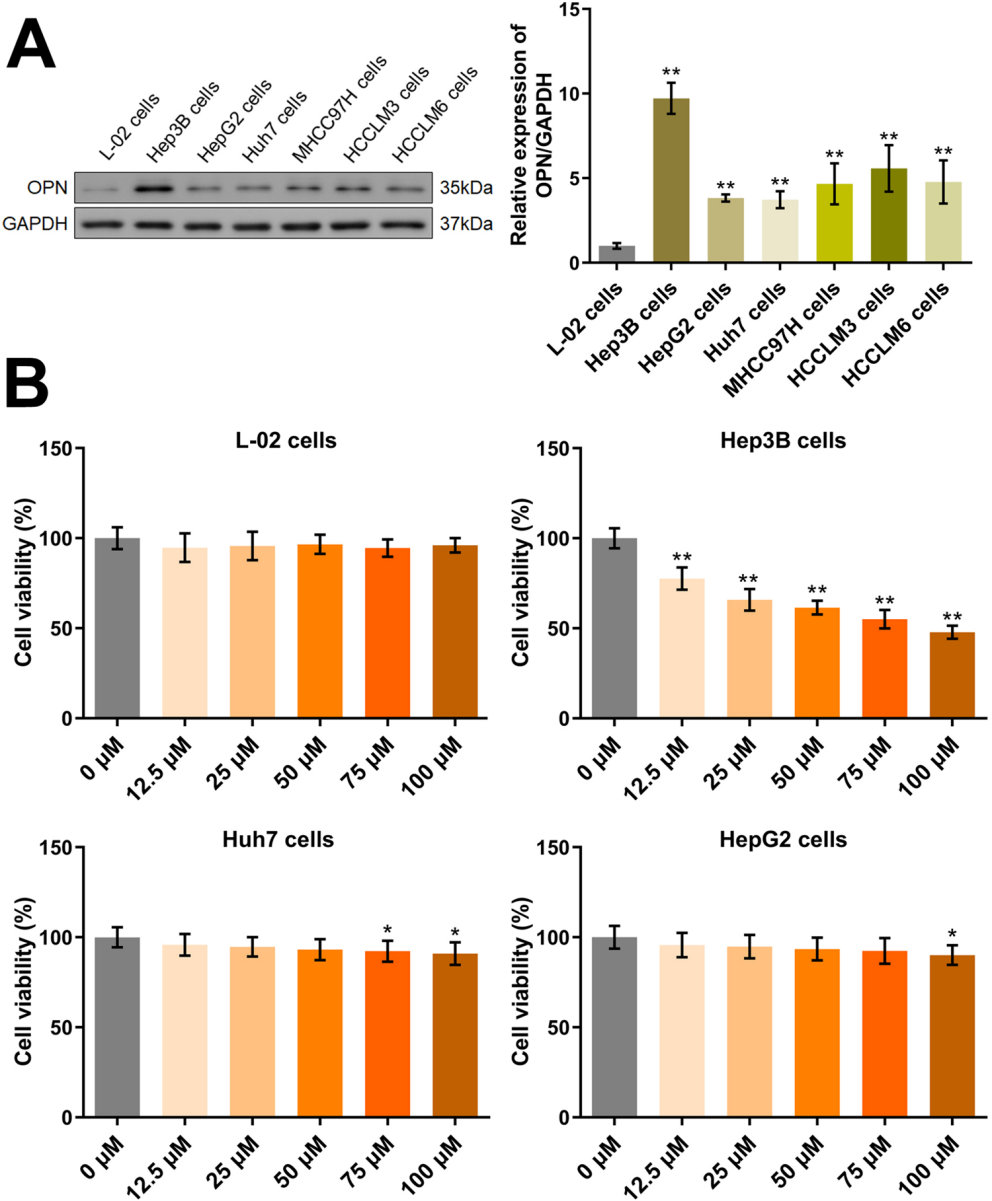
The HCC cell line and the concentration of fenofibrate were determined. **A** The expression level of OPN in L-02 cells and HCC cells was detected by the Western blotting assay (**p<0.01 vs. L-02 cells). **B** The cell viability in L-02 cells and HCC cells was detected by CCK-8 assay (*p<0.05 vs. 0 μM, **p<0.01 vs. 0 μM)

Firstly, the level of OPN in normal hepatocytes and 6 HCC cell lines was determined. Compared to L-02 cells, OPN was found extremely upregulated in 6 HCC cell lines, among which the highest expression of OPN was observed in Hep3B cells (Fig. 1A). Furthermore, to determine the concentration of fenofibrate and the target HCC cell line, cells were treated with 0, 12.5, 25, 50, 75, and 100 μM fenofibrate, followed by evaluating the cell viability using the CCK-8 assay (Fig. 1B). In L-02 cells, no impact of fenofibrate on the cell viability at all concentrations was observed. In Hep3B cells and Huh7 cells, the cell viability was signally repressed by fenofibrate in a concentration-dependent manner. However, in HepG2 cells, minor changes on the cell viability were observed by fenofibrate. Collectively, Hep3B cells and Huh7 cells, as well as 75 and 100 μM fenofibrate, were applied in subsequent assays.

### Fenofibrate inhibited the proliferation and migration, and facilitated the apoptosis of HCC cells by downregulating OPN

**Fig. 2:**
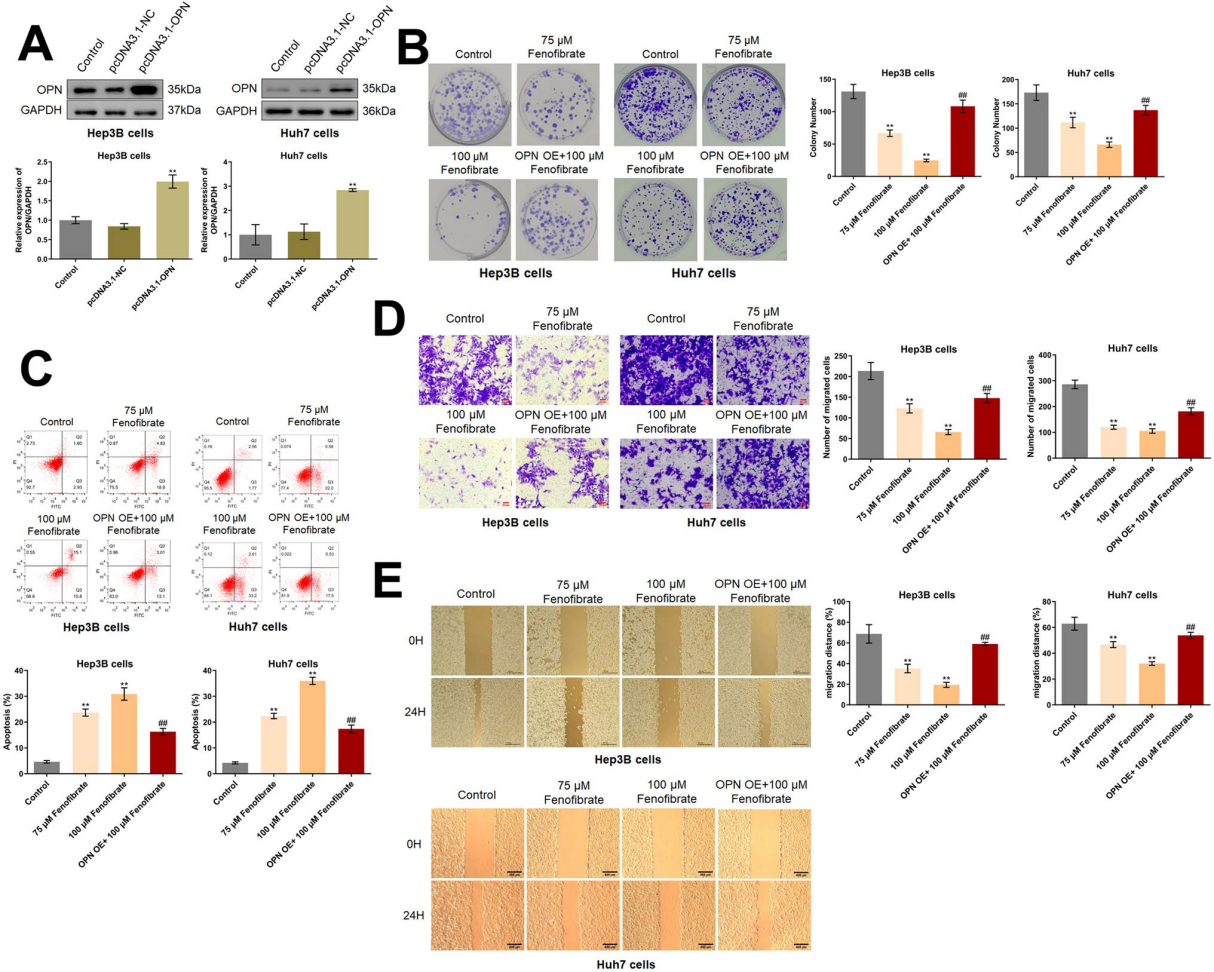
Fenofibrate inhibited the proliferation and migration, and facilitated the apoptosis of Hep3B cells and Huh7 cells by downregulating OPN. **A** The expression level of OPN was detected by the Western blotting assay (**p<0.01 vs. pcDNA3.1-NC). **B** The growth of Hep3B cells and Huh7 cells was evaluated by the clone formation assay. **C** The apoptotic rate was detected by the flow cytometry. **D** The migration of Hep3B cells and Huh7 cells was measured by the Transwell assay. **E** The wound healing assay was utilized to evaluate the migration ability of Hep3B cells and Huh7 cells (**p<0.01 vs. control, ## *p*<0.01 vs. 100 μM fenofibrate)

To obtain OPN-overexpressed cells, Hep3B cells and Huh7 cells were transfected with adenovirus containing pcDNA3.1-OPN, with pcDNA3.1-NC as a negative control. Compared to pcDNA3.1-NC, OPN was found dramatically upregulated in the pcDNA3.1-OPN groups (Fig. 2A), suggesting the successful establishment of OPN-overexpressed Hep3B cells and Huh7 cells. Subsequently, HCC cells were treated with 75 and 100 μM fenofibrate for 24 h, while OPN-overexpressed HCC cells were treated with 100 μM fenofibrate for 24 h. In Hep3B cells, the colony number was dramatically repressed from 131.0 to 66.7 and 24.7 by 75 and 100 μM fenofibrate, respectively. Compared to 100 μM fenofibrate, the colony number was reversed to 108.3 by the overexpression of OPN. In Huh7 cells, the colony number was dramatically repressed from 173.0 to 111.7 and 66.0 by 75 and 100 μM fenofibrate, respectively. Compared to 100 μM fenofibrate, the colony number was reversed to 137.0 by the overexpression of OPN (Fig. 2B). Furthermore, in Hep3B cells, the apoptotic rate in the control, 75 μM fenofibrate, 100 μM fenofibrate, and OPN OE+ 100 μM fenofibrate groups was 4.62%, 23.69%, 30.88%, and 16.29%, respectively. In Huh7 cells, the apoptotic rate in the control, 75 μM fenofibrate, 100 μM fenofibrate, and OPN OE+ 100 μM fenofibrate groups was 4.20%, 22.39%, 35.98%, and 17.37%, respectively (Fig. 2C). In Hep3B cells, the number of migrated cells was markedly reduced from 213.3 to 123.0 and 65.7 by 75 and 100 μM fenofibrate, respectively. Compared to 100 μM fenofibrate, the number of migrated cells was reversed to 148.0 by the overexpression of OPN. In Huh7 cells, the number of migrated cells was markedly reduced from 286.0 to 120.0 and 105.3 by 75 and 100 μM fenofibrate, respectively. Compared to 100 μM fenofibrate, the number of migrated cells was reversed to 181.3 by the overexpression of OPN (Fig. 2D). Moreover, in Hep3B cells, the migration distance observed in the wound healing assay in the control, 75 μM fenofibrate, 100 μM fenofibrate, and OPN OE+ 100 μM fenofibrate groups was 68.8%, 35.3%, 19.5%, and 59.1%, respectively. In Huh7 cells, the migration distance observed in the wound healing assay in the control, 75 μM fenofibrate, 100 μM fenofibrate, and OPN OE+ 100 μM fenofibrate groups was 62.9%, 46.7%, 32.0%, and 53.9%, respectively (Fig. 2E). A dramatically inhibitory effect of fenofibrate on the in vitro growth and migration of HCC cells was observed, which might be mediated by the downregulation of OPN.

### Fenofibrate suppressed the PI3K/AKT/Twist pathway in Hep3B cells by downregulating OPN

**Fig. 3:**
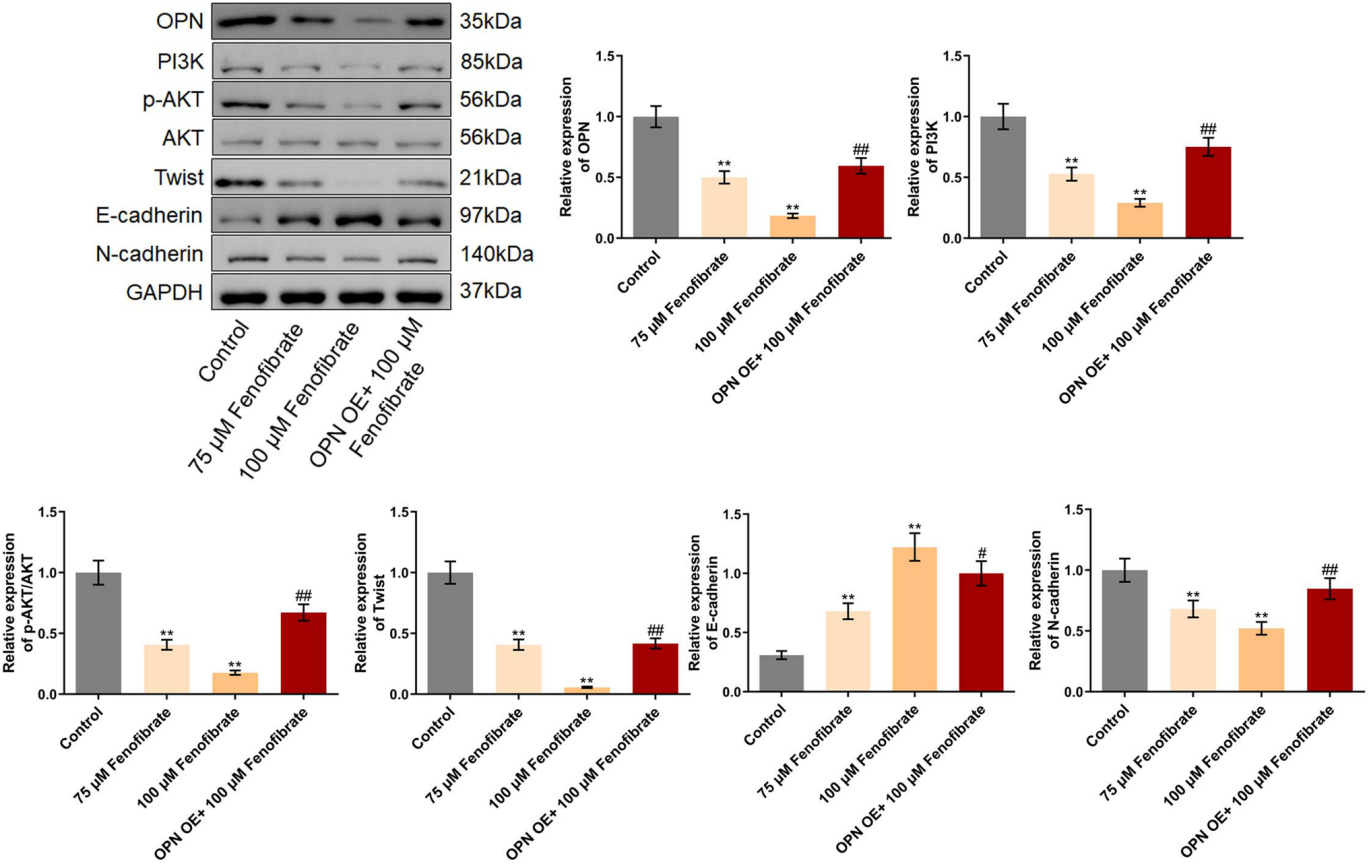
The PI3K/AKT/Twist pathway in Hep3B cells was suppressed by fenofibrate through downregulating OPN. The expression level of OPN, PI3K, p-AKT, AKT, Twist, E-cadherin, and N-cadherin in Hep3B cells was evaluated by the Western blotting assay (**p<0.01 vs. control, # *p*<0.05 vs. 100 μM fenofibrate, ## *p*<0.01 vs. 100 μM fenofibrate)

We further checked the function of fenofibrate on the PI3K/AKT/Twist pathway in Hep3B cells. Firstly, the level of OPN was found greatly suppressed by 75 μM and 100 μM fenofibrate, which was greatly reversed by the introduction of pcDNA3.1-OPN (Fig. 3). Furthermore, PI3K, p-AKT/AKT, Twist, and N-cadherin were found extremely downregulated, while E-cadherin was extremely upregulated by 75 μM and 100 μM fenofibrate. Compared to 100 μM fenofibrate, the level of PI3K, p-AKT/AKT, Twist, and N-cadherin was signally elevated, while the E-cadherin level was greatly decreased by the overexpression of OPN.

### The inhibitory function of fenofibrate on Hep3B cells was abolished by the agonist of PI3K

**Fig. 4:**
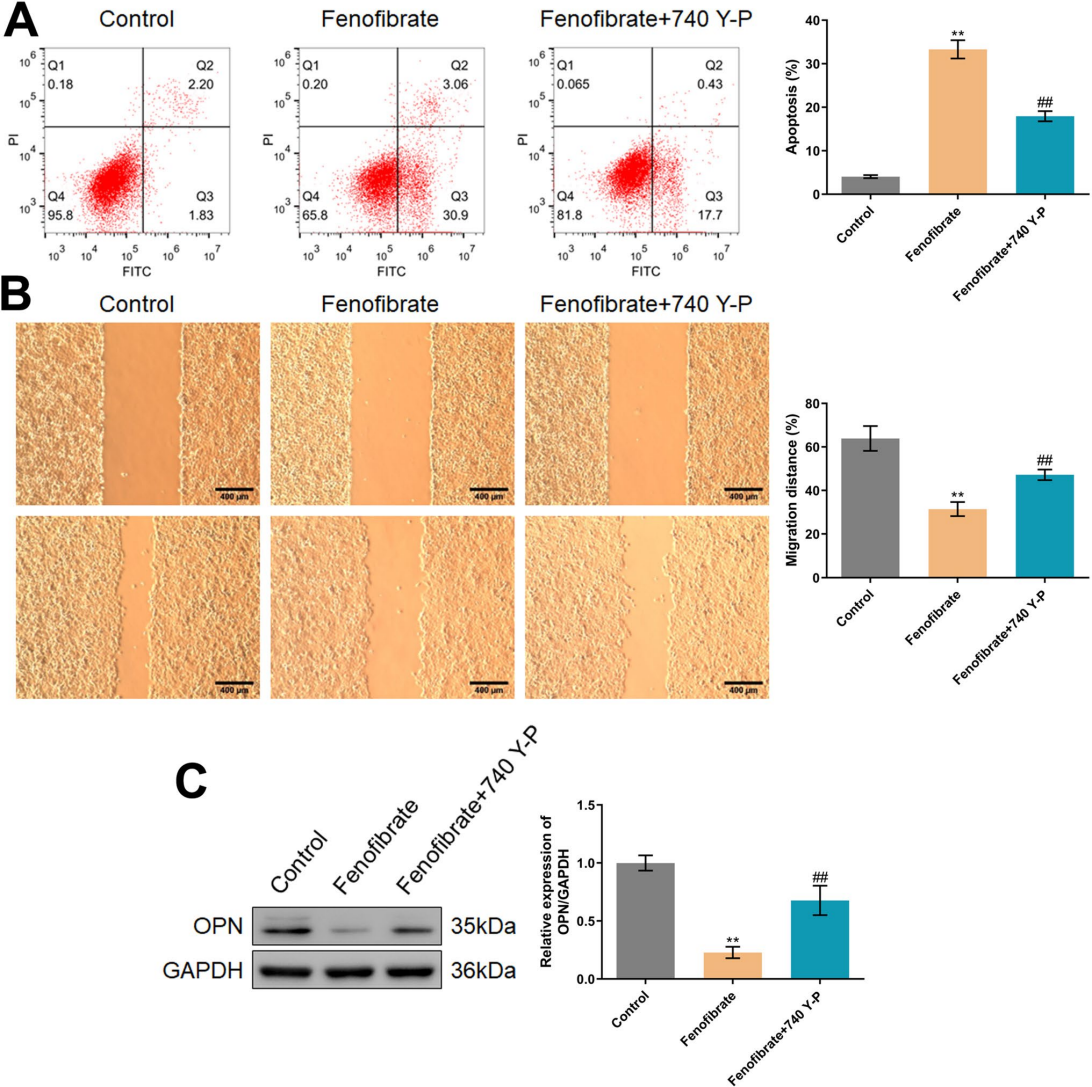
The inhibitory function of fenofibrate on Hep3B cells was abolished by the agonist of PI3K. **A** The apoptosis was determined by the flow cytometry. **B** The migration ability was checked by the wound healing assay. **C** The expression of OPN was detected by the Western blotting assay (**p<0.01 vs. control, ## *p*<0.01 vs. fenofibrate)

To confirm whether the regulatory function of fenofibrate in Hep3B cells was associated with the PI3K/AKT/Twist pathway, Hep3B cells were treated with 100 μM fenofibrate in the presence or absence of 10 μM 740 Y-P. The apoptotic rate of Hep3B cells was found markedly increased from 4.03 to 33.96%, which was greatly reduced to 18.13% by the co-culture of 740 Y-P (Fig. 4A). Furthermore, the migration distance in the control, fenofibrate, and fenofibrate+740 Y-P groups was 63.93%, 31.44%, and 47.23%, respectively (Fig. 4B). Moreover, the repressed OPN level observed in fenofibrate-treated Hep3B cells was found signally increased by the co-culture of 740 Y-P (Fig. 4C).

### Fenofibrate inhibited the in vivo growth of Hep3B cells by downregulating OPN

**Fig. 5:**
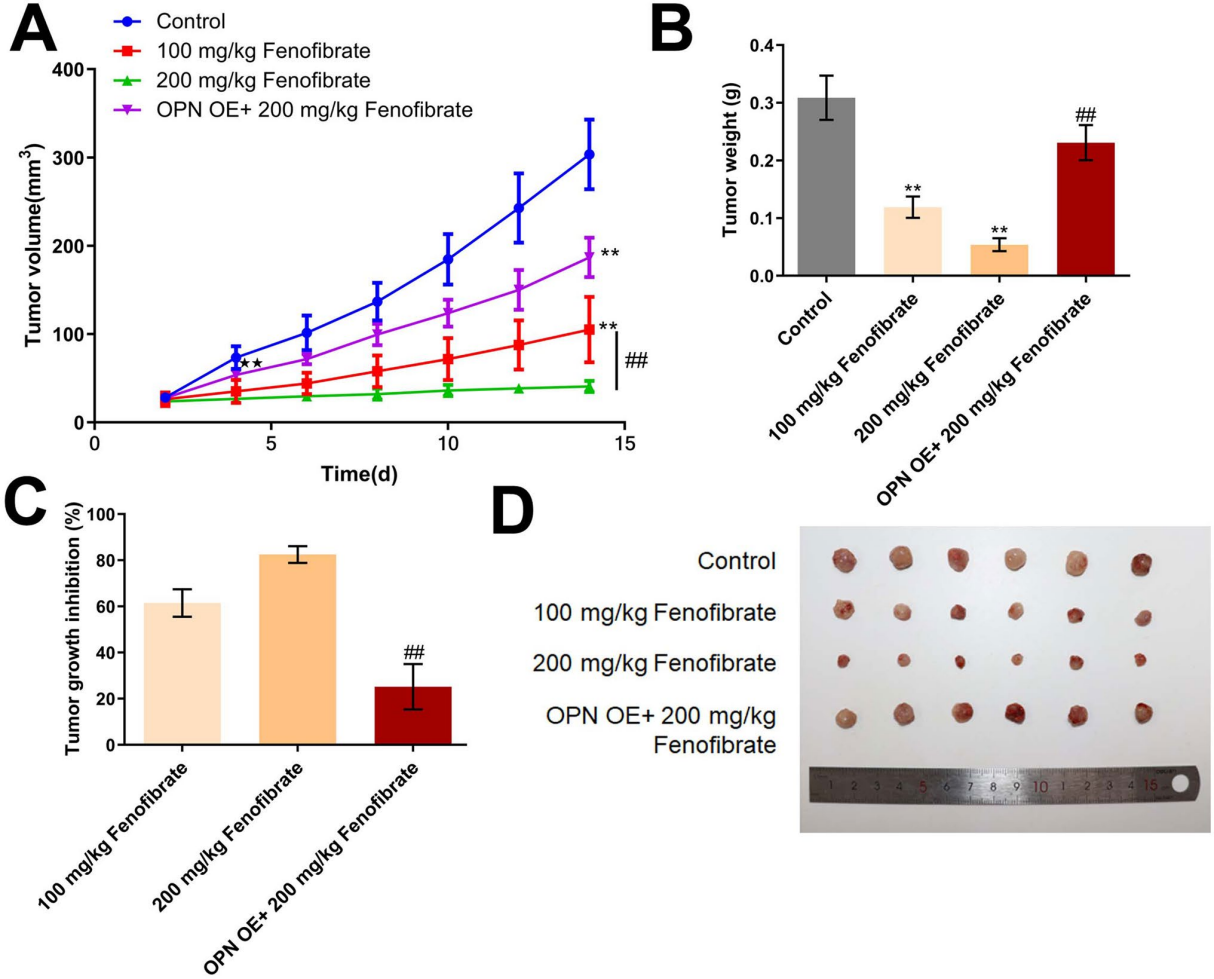
The in vivo growth of Hep3B cells was inhibited by fenofibrate via downregulating OPN. **A** The curve of tumor volume during the experiments was drawn. **B** The tumor weight at the end of the experiment was weighed. **C** The inhibitory rate in each group against Hep3B xenograft model was calculated (**p<0.01 vs. control, ## *p*<0.01 vs. 100 μM fenofibrate). **D** Images of tumor tissues were presented

To verify the anti-tumor property of fenofibrate, the xenograft model was constructed. We found that the tumor volume was dramatically suppressed by 100 mg/kg and 200 mg/kg fenofibrate. Compared to the 200 mg/kg fenofibrate group, the tumor volume was extremely reversed by the overexpression of OPN (Fig. 5A). Furthermore, the tumor weight in the control, 100 mg/kg fenofibrate, 200 mg/kg fenofibrate, and OPN OE+ 100 mg/kg fenofibrate groups was 0.31 g, 0.12 g, 0.05 g, and 0.23 g, respectively (Fig. 5B). The tumor growth inhibition rate in the 100 mg/kg fenofibrate and 200 mg/kg fenofibrate was 61.50% and 82.51%, respectively. Compared to the 200 mg/kg fenofibrate group, the tumor growth inhibition rate was decreased to 25.22% by the overexpression of OPN (Fig. 5C). Images of tumors were shown in Fig. 4D.

### Fenofibrate suppressed the PI3K/AKT/Twist pathway in tumor tissues by downregulating OPN

**Fig. 6:**
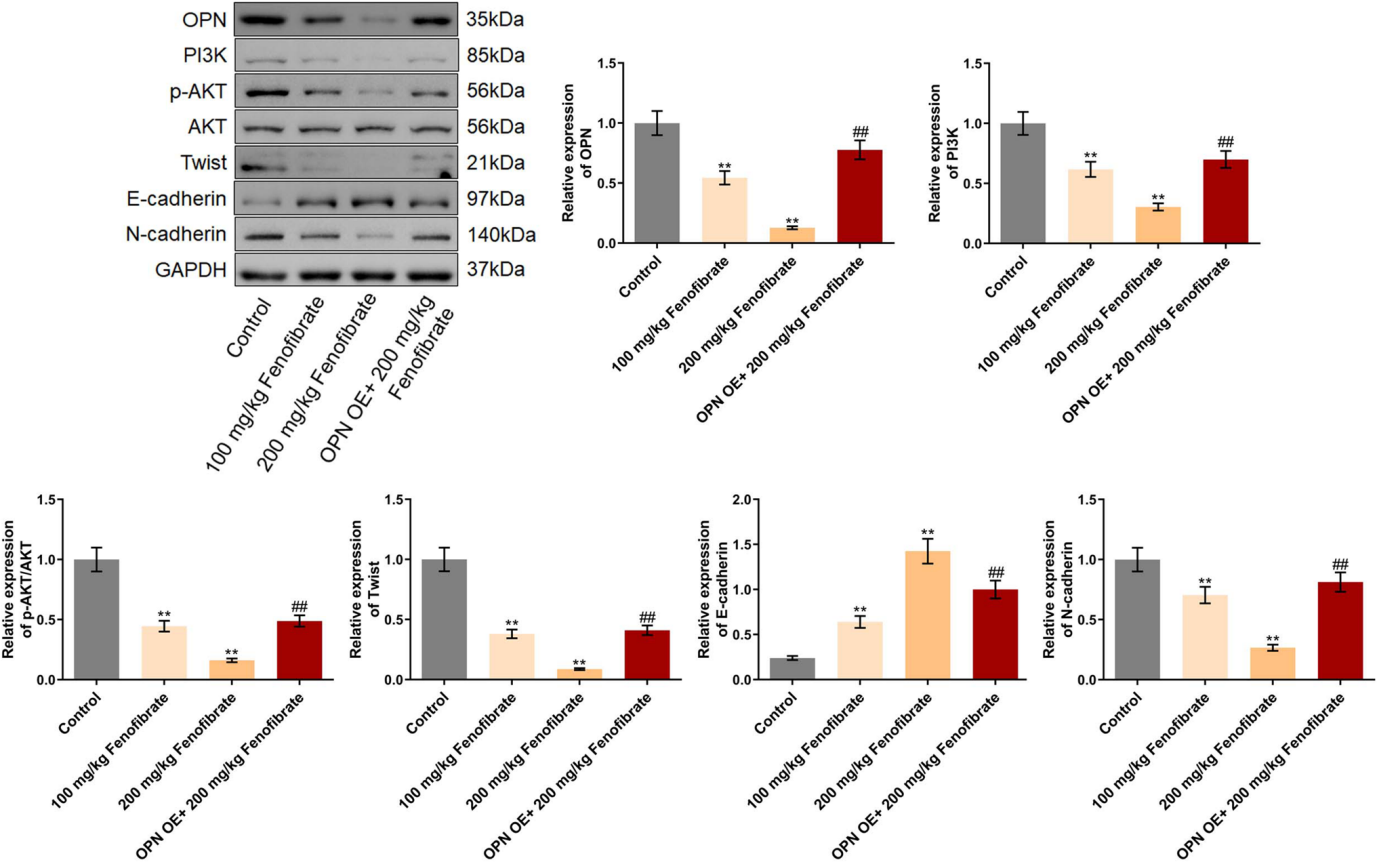
The PI3K/AKT/Twist pathway in tumor tissues was suppressed by fenofibrate through downregulating OPN. The expression level of OPN, PI3K, p-AKT, AKT, Twist, E-cadherin, and N-cadherin in Hep3B cells was evaluated by the Western blotting assay (**p<0.01 vs. control, ## *p*<0.01 vs. 100 μM fenofibrate)

Lastly, the regulatory mechanism of fenofibrate was verified in tumor tissues. OPN, PI3K, p-AKT/AKT, Twist, and N-cadherin were found signally downregulated, while E-cadherin was upregulated in tumor tissues by 100 mg/kg and 200 mg/kg fenofibrate. Compared to the 200 mg/kg fenofibrate group, the level of OPN, PI3K, p-AKT/AKT, Twist, and N-cadherin was signally elevated, while the E-cadherin level was markedly decreased by the overexpression of OPN (Fig. 6).

## Discussion

PHC is one of the most common malignant tumors of the digestive system in the world. The prevalence is relatively high in Asia and parts of the world, and the incidence is also increasing in Africa and Western countries (Chidambaranathan-Reghupaty et al. 2021). Most patients with PHC are treated with surgical resection, including liver transplantation, while standard chemotherapy and radiotherapy have limited efficacy. High invasiveness and metastasis are two major characteristics of PHC, resulting in poor prognosis even after surgical resection. The key to the treatment and prognosis of PHC is to inhibit and reduce the invasion and metastasis of liver cancer (Haber et al. 2021). In our study, the proliferation and migration of Hep3B cells was extremely suppressed by fenofibrate, accompanied by an elevation of apoptotic rate, which was in accordance with the performance of fenofibrate in breast cancer cells (Li et al. 2014), ovarian cancer cells (Wang et al. 2014), and pancreatic cancer cells (Hu et al. 2016). Furthermore, the in vivo xenograft model further confirmed the anti-tumor property of fenofibrate against HCC, which was also observed in PC-3 xenograft model (Tao et al. 2018).

OPN is a secreted phosphorylated glycoprotein that is widely distributed in human tissues and exerts various functions such as mediating cell adhesion, promoting neovascularization, and inhibiting cell apoptosis. It is proved that OPN is closely related to the occurrence, development, metastasis, and recurrence of multiple malignant tumors (Coppola et al. 2004). Lin et al. (Lin et al. 2013) claimed that OPN was largely synthesized and secreted in malignant tumor cells, especially in HCC. Therefore, in recent years, the relationship between OPN and the progression of HCC has become a hot topic for researchers. By using quantitative PCR, Gotoh et al. (Pan et al. 2003) found that the expression level of OPN in HCC tissues was significantly higher than that in normal liver tissues. Furthermore, the positive expression of OPN in the surrounding cells of tumor nodules is extremely significant. Pan et al. (Pan et al. 2003) found that elevated AFP, p53 mutation, large tumor size, late stage, high grade, early recurrence or metastasis, and low 10-year survival rate were closely related to the high mRNA level of OPN in HCC patients. Moreover, in some patients with early HCC, the high mRNA level of OPN plays a role in predicting early recurrence. In our study, a critical upregulation of OPN was observed in 3 HCC cell lines, which was consistent with previous reports (Wu et al. 2022). Furthermore, a close relationship between the expression of OPN and the suppressive effect of fenofibrate on the cell viability was observed in 3 HCC cell lines, implying that the anti-tumor property of fenofibrate might be associated with OPN. Both in Hep3B cells and tumor tissues of Hep3B cell xenograft model, OPN was found extremely downregulated by fenofibrate, which was in line with previous researches (Rowbotham et al. 2018; Moxon et al. 2020). Moreover, the in vitro and in vivo anti-tumor function of fenofibrate were markedly abolished by the overexpression of OPN, suggesting that the function of fenofibrate was mediated by OPN.

Twist was first identified in Drosophila in 1983, which is a highly conserved basic helix-loop-helix-DNA binding transcription factor that specifically regulates the expression of key target genes. Twist was initially found to regulate embryonic development by promoting epithelial-mesenchymal transition (EMT) during embryonic development (Jin et al. 2020). With the continuous in-depth study of the biological mechanism of EMT, it has been found that Twist, as a key regulator in EMT, is closely related to tumor invasion and metastasis (Yu et al. 2012), which is found to inhibit the E-cadherin promoter to result in EMT (Vermani et al. 2020). Existing studies have shown that the signaling pathway of Twist is a multi-step, multi-pathway, and multi-level network association structure, and a variety of important signal transduction pathways are related to Twist, including PI3K/Akt axis. Li et al. (Li and Zhou 2011) studied the expression of Twist in MCF-7 cells and Hela cells and found that the high expression of Twist induced the morphological changes of EMT, accompanied by the activation of Akt and β-catenin signaling pathways. In our study, the anti-tumor property of fenofibrate was accompanied by an inhibition of PI3K/AKT/Twist signaling and EMT progression, which was also observed in fenofibrate treated renal transplant model (Wang et al. 2019). Furthermore, the regulatory effect of fenofibrate on the PI3K/AKT/Twist signaling and EMT progression was abolished by the overexpression of OPN, implying that OPN was a key mediator involved in the regulatory mechanism of fenofibrate. In future work, the regulatory mechanism of fenofibrate will be further verified by co-treating HCC cells with fenofibrate and an agonist of Twist.

Collectively, fenofibrate suppressed progression of hepatoma downregulating OPN through inhibiting the PI3K/AKT/Twist pathway.

## Data Availability

The datasets used and/or analyzed during the current study are available from the corresponding author on reasonable request.
